# Exendin-4 — A potential therapeutic for type 2 diabetes-linked cervical cancer?

**DOI:** 10.1016/j.ebiom.2021.103273

**Published:** 2021-03-11

**Authors:** Nivida Mishra, Suresh Mishra

**Affiliations:** a Faculty of Engineering, University of Manitoba, Manitoba, Winnipeg, Canada; b Department of Internal Medicine, Faculty of Health Sciences, University of Manitoba, Manitoba, Winnipeg, Canada

Accruing evidence suggests that type 2 diabetes (T2D) increases the risk of many cancer types and cancer-associated mortality at large. Hyperglycaemia, manifesting in relation to T2D, appears to play an important role in this trend of increased risk. In addition, co-existing inflammations might further enhance the incidence of T2D-linked cancer [1]. However, our understanding of the underlying mechanisms in these relationships remains poor. Uncontrolled cell proliferation is a hallmark of cancer development, which requires high protein turnover in cancer cells and involves the proteasome system [2]. Notably, the proteasome system is also dysregulated in T2D [3], raising the possibility for it to play a potential role in T2D-linked cancer. In an article published in *EBioMedicine*, Mao *et al* show an increased co-expression of the proteasome alpha 2 subunit (*PSMA2*, a proteasome gene) and the glucagon-like peptide-1 receptor (*GLP-1R*) in cervical cancer models in T2D, which is attenuated by Exendin-4, a GLP-1R agonist [4].

Exendin-4, which is a GLP-1 mimetic, is a new class of blood glucose-lowering drugs, which binds with GLP-1R to perform its job in T2D patients [5]. Emerging evidence suggests that this GLP-1 mimetic might also have an anti-cancer effect. For instance, a meta-analysis of randomized, controlled trials involving 63 594 patients with T2D indicated that treatment with albiglutide (a GLP-1 agonist) was associated with a 24% reduction of all sites of cancer when compared to the placebo group [6]. Moreover, in prostate cancer patients, Exendin-4 has been shown to enhance the responsiveness to chemotherapy and reduce cancer growth [7]. In addition to its blood glucose-lowering effect, the GLP-1 mimetic exerts an anti-inflammatory effect on a variety of tissues and organs in the body [8]. As inflammation plays a key role in the development of many cancer types, it is possible that this understated anti-cancer effect of the GLP-1 mimetic is linked to its anti-inflammatory effect. Of note, the GLP-1 mimetic is reported to inhibit pancreatic and breast cancer cell proliferation through the inhibition of the NF-κB pathway in experimental studies [7,9].

In this case-control study, Mao et al. [4] have used a combination of complementary experimental designs and approaches. This includes the exploration of the Cancer Genome Atlas (TCGA) and the Genotype-Tissue Expression (GTEx) databases for tumour-specific and normal tissue-specific expression analyses of *PSMA2*, expression analyses of *PSMA2* and *GLP-1R* levels in cervical specimens from patients with and without T2D, and subsequent validation of the results *in vivo* using preclinical mouse models and *in vitro* using cervical cancer cell models. Thus, the authors provided convincing evidence to support their findings and conclusions on numerous levels.

As proteasome inhibitors are approved drugs for the treatment of multiple myeloma and lymphoma, and GLP-1R agonists for the treatment of T2D, the translational potential of these research findings is high. However, a number of pertinent questions remain unanswered. For example, whether the relationship between *PMSA2* and *GLP-1R* expression is specific to cervical cancer, or common across other T2D-related cancer types, remains unclear. Moreover, the underpinnings of why high glucose levels lead to an upregulation of *PMSA2* in cancer cells and not in normal cells (a cause or a consequence of the T2D-linked cervical cancer) has not presently been investigated. The effects of high glucose levels were observed only in the CUP-1 cells (modeled murine epithelial cervical cancer cells), but not in non-cancerous mouse fibroblast and human embryonic kidney cells, which would imply these effects to be a feature of cancer cells. Thus, understanding the mechanisms involved in the effect of high glucose levels on *PMSA2* expression is of clinical significance. In addition, whether the effect of Exendin-4 on reducing *PSMA2* expression level *in vivo* is mediated indirectly through its glucose-lowering function, or directly at the tissue level, also remains unclear. In this context, it should be noted that the knockdown of *GLP-1R* in human and mouse cervical cancer cell lines led to the corresponding downregulation of *PSMA2*, implying a direct effect, independent of the glucose-lowering function of Exendin-4. Moving forward, it will be important to unravel the basic mechanisms involved in the relationship between the co-expression of *PSMA2* and *GLP-1R* in T2D-linked cervical cancer, and their downregulation by Exendin-4. The *db/db* mouse model (which carry a genetic mutation in the leptin receptor and develop T2D because of hyperphagia-linked obesity), which was used for the *in vivo* validation study, may not accurately represent the patient cohort used in this study. This would require further validation using other, more relevant preclinical models of T2D [10].

Another potential avenue that needs further exploration includes the anti-inflammatory effect of Exendin-4 that has been reported in experimental studies. Thus, an investigation of the crosstalk between hyperglycaemia and inflammation may provide additional insights into the beneficial effects of Exendin-4 in T2D-linked cervical cancer. Thus, a major question that remain unanswered is how the mechanistic link between GPL-1R and PSMA2 in T2D-linked cervical cancer works, and the relative contribution of both T2D-related hyperglycaemia and inflammation therein. Other limitations that are also pointed by the authors include a selection bias, since only samples from Chinese women were included and other confounding factors were not adjusted, such as previous treatment or other risk factors. Despite these limitations and unanswered pertinent questions, this study by Mao *et al* sets the foundation for future clinical trials to determine the anti-cancer effects of Exendin-4 for T2D-linked cervical cancer (and potentially other T2D-linked cancer types) and for basic research to unravel the underlying mechanisms involved.

## Contributors

NM and SM wrote and edited the commentary. SM did the literature search used for the commentary.

## Declaration of Competing Interest

The authors do not have conflict of interest.
